# Pyridocarbene‐Based Tetradentate Pt(II) Complexes for Long Device Lifetime over 500 h in Blue Phosphorescent Organic Light–Emitting Diodes

**DOI:** 10.1002/adma.202510070

**Published:** 2025-08-19

**Authors:** Kiun Cheong, Hyunjung Lee, Jangho Moon, Chan Hee Ryu, Gyeong Woo Kim, Jun Yun Kim, In‐Ho Lee, Yong‐Woo Kim, Young Woong Lee, Seokhyeon Yu, Kang Mun Lee, Sunwoo Kang, Jun Yeob Lee

**Affiliations:** ^1^ School of Chemical Engineering Sungkyunkwan University 2066, Seobu‐ro, Jangan‐gu Suwon‐si Gyeonggi 16419 Republic of Korea; ^2^ Department of Display Convergence Engineering Sungkyunkwan University 2066, Seobu‐ro, Jangan‐gu Suwon Gyeonggi 16419 Republic of Korea; ^3^ Department of Chemistry Institute for Molecular Science and Fusion Technology Kangwon National University Chuncheon Gangwon 24341 Republic of Korea; ^4^ LG Display Co. Ltd. LG Science Park, 30, Magokjungang 10‐ro, Gangseo‐gu Seoul 07796 Republic of Korea; ^5^ P&H Tech 16–25, Dongbaekjungang‐ro 16beon‐gil, Giheung‐gu Yongin‐si Gyeonggi 17015 Republic of Korea; ^6^ LT Materials 113–19, Dangha‐ro, Namsa‐eup, Cheoin‐gu Yongin‐si Gyeonggi 17118 Republic of Korea; ^7^ Department of Chemistry Dankook University Cheonan Chungnam 31116 Republic of Korea; ^8^ SKKU Institute of Energy Science and Technology Sungkyunkwan University 2066, Seobu‐ro, Jangan‐gu Suwon Gyeonggi 16419 Republic of Korea

**Keywords:** organic light–emitting diode, phosphorescence, Pt(II) complex

## Abstract

Blue phosphorescent organic light–emitting diodes (PhOLEDs) face challenges in achieving high efficiency, color purity, and long device lifetime due to exciton quenching and high energy requirements. In this study, two tetradentate Pt(II) complexes, **Pt‐impy** and **Pt‐Me‐impy**, are designed and synthesized by incorporating pyridocarbene in their ligands. Pyridocarbene enhances the electrochemical stability, strengthens triplet metal‐to‐ligand charge transfer characteristics, and improves the spin–orbit coupling, effectively shortening the exciton lifetime and minimizing the quenching effects. Additionally, the introduction of a methyl group as a conformation manager in **Pt‐Me‐impy** further improves the color purity by blue‐shifting and narrowing the emission spectrum. As a result, **Pt‐impy** and **Pt‐Me‐impy** exhibit external quantum efficiencies of 24.5% and 27.4% at the 3 wt.% doping concentration, respectively. In particular, **Pt‐impy** and **Pt‐Me‐impy** doped blue PhOLEDs optimized for stability demonstrate a device lifetime of up to 95% of the initial luminance of 543 and 228 h at 1,000 cd m^−2^, which has never been achieved in the blue PhOLEDs. This study demonstrates that the synergistic combination of pyridocarbene and the methyl conformation manager is an effective strategy for improving blue PhOLED performance, enhancing their potential for practical applications.

## Graphical abstract

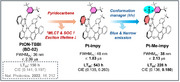

## 1 Introduction

Phosphorescent emitters utilize all excitons generated during the electroluminescence (EL) process for light emission by enhancing the spin‐orbit coupling (SOC) through the heavy atom effect of the central metal and the mixing of singlet‐triplet excitons.^[^ ^1^ ^]^ This results in 100% internal quantum efficiency in phosphor‐based organic light–emitting diodes (OLEDs), surpassing that of the fluorescent OLEDs. This has been demonstrated by their successful incorporation into commercialized red and green OLEDs. For blue OLEDs, although phosphorescent emitters with high color purity and high efficiency are emerging, their high emission energy poses challenges for developing stable materials for long device lifetime.^[^ ^2^ ^]^ To address the stability issue, it is necessary to realize a narrowband emission spectrum that reduces short‐wavelength components without compromising the pure blue emission color. Furthermore, the decay rate of the triplet excitons must be increased to minimize the triplet–triplet annihilation (TTA) and triplet‐polaron annihilation (TPA) mechanisms, which are critical to the device lifetime.^[^ ^3^ ^]^

Tetradentate Pt(II) complexes possess structural stability due to the coordination of a single rigid ligand to the Pt metal and exhibit strong performance as emitters in phosphorescent OLEDs (PhOLEDs).^[^ ^4^ ^]^ In particular, structures containing 5/6/6 metallacycles break the square‐planar conformation and effectively suppress intermolecular interactions and metal–metal‐to‐ligand charge transfer (MMLCT).^[^ ^5^ ^]^ Additionally, unlike Ir(III) phosphors, the low metal‐to‐ligand charge transfer (MLCT) contribution to the lowest triplet excited state (T_1_) induces triplet ligand‐centered (^3^LC) emission, resulting in a narrow spectral bandwidth with distinct vibrational peaks.^[^ ^6^ ^]^ However, the reduced metal contribution to the T_1_ leads to a slower decay rate due to weaker SOC.^[^ ^1^, ^7^ ^]^ In other words, to improve the operational device lifetime of tetradentate Pt(II) complexes in PhOLEDs, it is essential to increase the metal contribution to the emission state, thereby enhancing MLCT characteristics and reducing exciton lifetimes.

To enhance SOC, the lowest unoccupied molecular orbital (LUMO) level of the ligand should be stabilized, and the metal contribution to the highest occupied molecular orbital (HOMO) should be increased to promote the probability of MLCT.^[^ ^8^ ^]^ Li et al. reported **PtON5‐dtb** and **PtON5N‐dtb**, which the N‐heterocyclic carbene (NHC) of **PtON7‐dtb** was replaced with benzocarbene and pyridocarbene, respectively.^[^ ^9^ ^]^ These modifications stabilized the LUMO level and enhanced MLCT by expanding the π‐conjugation using pyridine. As a result, **PtON5N‐dtb** exhibited a longer device lifetime than **PtON7‐dtb** in PhOLEDs. It has high PLQY and short exciton lifetime, but it still shows a broad emission spectrum and red‐shifted wavelength.

In this study, platinum(II) 1‐(3‐((9‐(4‐(*tert*‐butyl)pyridin‐2‐yl*‐κ*N)‐9*H*‐carbazol‐2‐yl‐*κ*C^1^)oxy)phenyl*‐κ*C^1^)‐3‐(3,5‐di‐*tert*‐butylphenyl)‐3*H*‐imidazo[4,5‐*b*]pyridin‐2‐ylidene‐*κ*C^2^ (**Pt‐impy**) was synthesized by replacing the benzocarbene in **PtON‐TBBI** (**BD‐02**) with pyridocarbene, for a long device lifetime,^[^ ^10^ ^]^ and platinum(II) 1‐(3‐((9‐(4‐(*tert*‐butyl)pyridin‐2‐yl*‐κ*N)‐9*H*‐carbazol‐2‐yl‐*κ*C^1^)oxy)phenyl*‐κ*C^1^)‐3‐(3,5‐di‐*tert*‐butylphenyl)‐4‐methyl‐3*H*‐imidazo[4,5‐*b*]pyridin‐2‐ylidene‐*κ*C^2^ (**Pt‐Me‐impy**) was developed by introducing a methyl group at 7‐position of the pyridocarbene as a conformation manager to improve the color purity while maintaining the improved device lifetime. The introduction of the pyridocarbene strengthened MLCT characteristics in the T_1_ and reduced the exciton lifetimes. Additionally, the methyl conformation manager blue‐shifted and sharpened the emission spectrum while enhancing the emission efficiency. As a result, **Pt‐impy** and **Pt‐Me‐impy** exhibited external quantum efficiencies (EQEs) of 24.5% and 27.4% at the 3 wt.% doping concentration, respectively. In particular, **Pt‐impy** and **Pt‐Me‐impy** doped blue PhOLEDs optimized for stability demonstrated device lifetimes up to 95% of the initial luminance (LT_95_) of 543 and 228 h at 1000 cd m^−2^, which have never been achieved in the blue PhOLEDs. This study confirmed that the combination of the pyridocarbene and methyl conformation manager is an effective approach to overcome the lifetime hurdle of blue PhOLEDs.

## 2 Results and Discussion

### 2.1 Design Concept and Synthesis

To increase the operational device lifetime of PhOLEDs, the long exciton lifetime of the phosphorescent emitters must be shortened. The effective way to achieve a shorter exciton lifetime is by strengthening the SOC. In general, the SOC of phosphors is closely associated with their MLCT character, which reflects the metal contribution to the emission state. Therefore, a design strategy that intensifies the MLCT character can enhance the SOC and reduce the triplet exciton lifetime of the Pt(II) phosphors. This ultimately helps suppress degradation processes, such as TTA and TPA, enabling the stable operation of PhOLEDs. One efficient approach to improving the SOC is to adjust the LUMO level of the tetradentate NHC ligand‐based Pt(II) complexes using the imidazo[4,5‐b]pyridine unit. Compared to benzimidazole, the imidazo[4,5‐b]pyridine unit contains an additional sp^2^ nitrogen atom and exhibits a relatively deeper LUMO level, making it an electron‐deficient heterocyclic unit. As a result, pyridocarbene derived from the imidazo[4,5‐b]pyridine unit may induce a deeper LUMO level than benzocarbene when used as the main framework of the tetradentate ligand in Pt(II) complexes.

**Figure 1:**
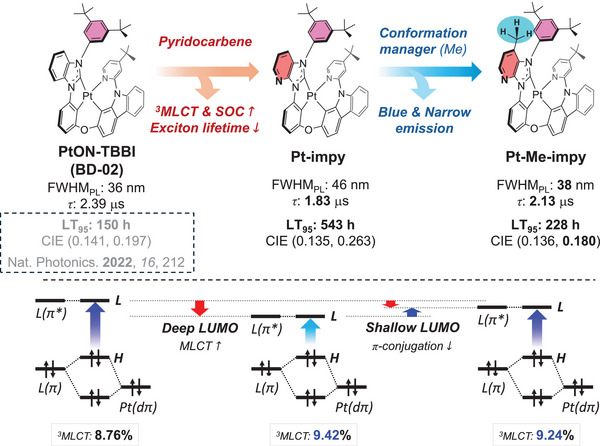
Design concept of **Pt‐impy** and **Pt‐Me‐impy**.

The **Pt‐impy** was designed with a pyridocarbene‐based tetradentate ligand to achieve a deeper LUMO level and accompanying increased ^3^MLCT character for stronger SOC. However, the HOMO–LUMO gap may become small because of the deeper LUMO level, and the emission spectrum can be red‐shifted, degrading the color purity of the blue PhOLEDs. Moreover, the increase in the charge transfer component increases the FWHM. To address these issues, **Pt‐Me‐impy** was developed by incorporating a methyl group as a conformation manager at the 7‐position of pyridocarbene. The methyl conformation manager may distort the 3,5‐di‐tert‐butylphenyl (dtp) N‐substituent linked to pyridocarbene through steric hindrance, thereby preventing π‐conjugation expansion. Additionally, the electron‐donating ability of the methyl group may weaken the electron deficiency of the pyridocarbene and adjust the LUMO level for widened HOMO–LUMO gap. As a result, the ^3^MLCT character would be weakened and the rigidity would be enhanced by the conformation manager, which may result in the blue‐shifted emission peak and sharpen the emission spectrum in **Pt‐Me‐impy**. This design concept is shown in **Figure** **1**.

**Figure 2:**
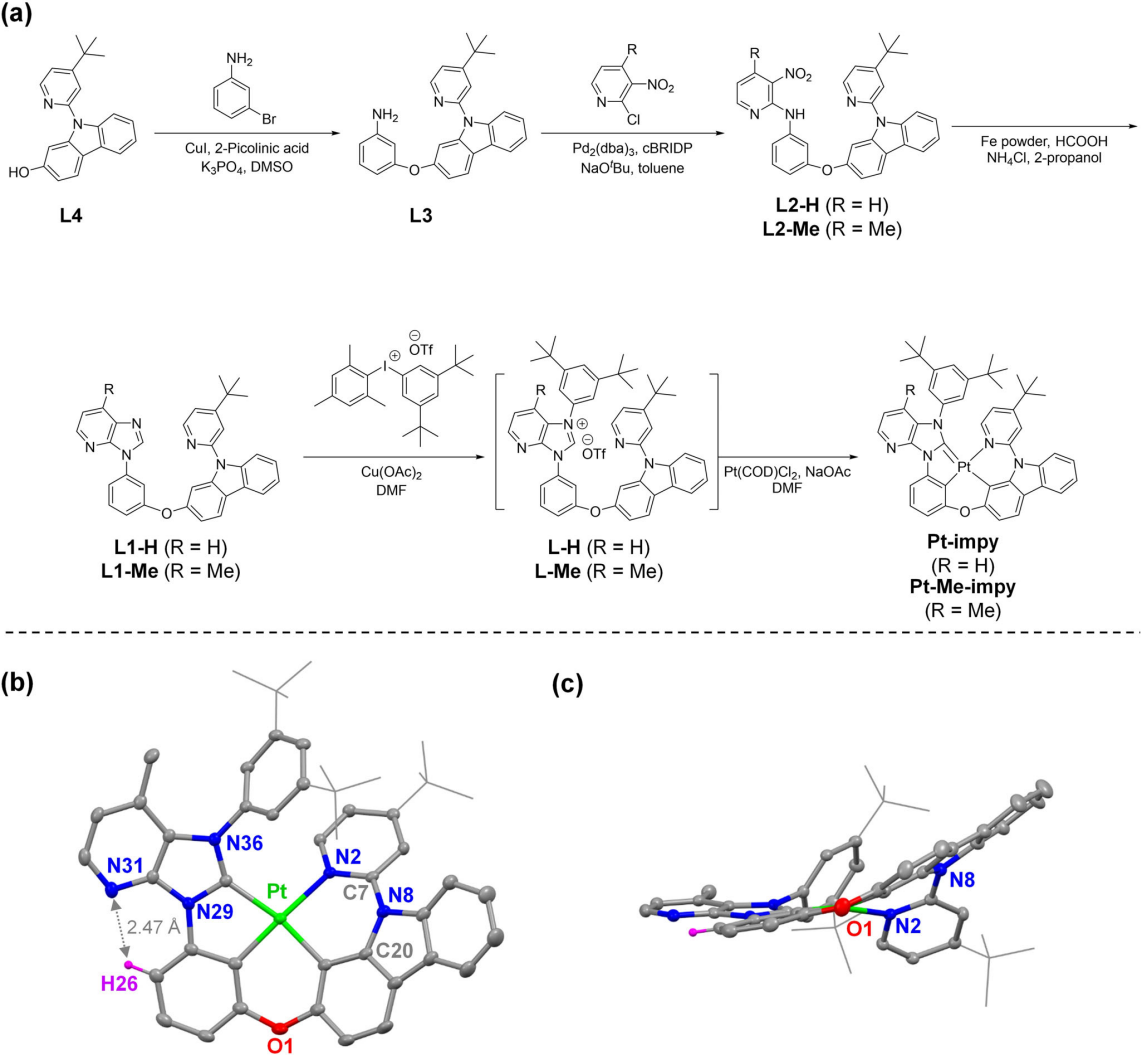
a). Synthetic process of **Pt‐impy** and **Pt‐Me‐impy**. b) Top and c) side views of the X‐ray crystal structure of **Pt‐Me‐impy** (thermal ellipsoids at 40% probability). For clarity, the H atoms (except for H26) are omitted. The solid lines represent the alkyl groups. Detailed crystallographic data are presented in Tables S1 and S2 (Supporting Information).

The ligands of Pt(II) complexes were synthesized, as shown in **Figure** **2**a. **L2‐H** and **L2‐Me** were synthesized from 3‐((9‐(4‐(*tert*‐butyl)pyridin‐2‐yl)‐9*H*‐carbazol‐2‐yl)oxy)aniline (**L3**) via Buchwald–Hartwig amination with 2‐chloro‐3‐nitropyridine and 2‐chloro‐4‐methyl‐3‐nitropyridine, respectively. Iron powder, formic acid, and ammonium chloride were used to synthesize the imidazo[4,5‐b]pyridine unit by reducing the nitro group and imidazole cyclization. To introduce the dtp unit from **L1‐H** and **L1‐Me**, **L‐H** and **L‐Me** were synthesized through a copper acetate catalytic reaction. Silica column chromatography was performed to improve the purity; however, the products were decomposed when methanol was used as the eluent. Therefore, short silica column filtration was performed using acetone as an eluent, yielding **L‐H** and **L‐Me**; however, the purity was not good. Therefore, the metalation reaction was performed from the crude product. As a result, **Pt‐impy** and **Pt‐Me‐impy** were obtained, and their structures were confirmed through ^1^H and ^13^C nuclear magnetic resonance spectroscopy and high‐resolution mass analysis (Supporting information).

The single‐crystal X‐ray diffraction analysis of **Pt‐Me‐impy** (Figure 2; Tables S1 and S2, Supporting Information) revealed key structural features, including the tetradentate coordination centered on Pt(II) metal atoms adopting a square‐planar geometry. The methyl substitution on the pyridocarbene moiety was clearly confirmed. Notably, the pyridine groups coordinated to each Pt(II) center exhibited significant distortion, which was attributable to the intramolecular steric hindrance imposed by the bulky 3,5‐di‐*tert*‐butylphenyl substituent. The angular sum around the Pt(II) center was measured at 362°, corroborating the geometric distortion. This distortion was further evidenced by the pronounced torsional angle (40°) observed for the N2–C7–N8–C20 moiety within the X‐ray structure. Additionally, the proximity of the N atom (N31) in the pyridocarbene unit to the H atom (H26) in the phenoxy group, measured at 2.47 Å, strongly suggests the presence of intramolecular hydrogen bonding. These structural characteristics indicate that the geometric flexibility and vibrational dynamics of the complex are significantly restricted by pronounced intramolecular steric hindrance and hydrogen bonding. Furthermore, crystal packing analysis within the unit cell revealed the absence of intermolecular π–π stacking and metal–metal interactions, as the Pt(II)–Pt(II) separation exceeded 4.5 Å (Figure S2, Supporting Information). This suggests a minimal propensity for aggregation or excimer formation through intermolecular interactions. In summary, the observed structural features and steric effects indicate that the geometric variation and vibrational motion of the Pt(II) complex are substantially constrained by severe intramolecular and intermolecular steric hindrance, as well as hydrogen bonding.

### 2.2 Photophysical and Electrochemical Properties

**Figure 3:**
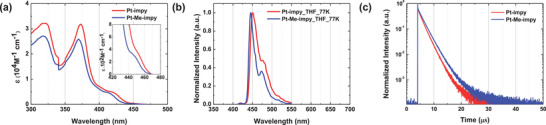
a) UV–vis absorption spectra, b) photoluminescence spectra at 77 K of **Pt‐impy** and **Pt‐Me‐impy**, and c) time‐resolved photoluminescence curves of **Pt‐impy** and **Pt‐Me‐impy** with the 3‐CzPB host.

The UV–vis absorption spectra of **Pt‐impy** and **Pt‐Me‐impy** were measured in a diluted methylene chloride (MC) solution (**Figure** **3**a). Strong ^1^LC (π–π*) absorption bands were recorded at a high energy of 340 nm, exhibiting high extinction coefficients. The ^1^MLCT absorption band appeared above 400 nm, whereas ^3^MLCT absorption, enabled by SOC, appeared around 440 nm with low extinction coefficients. **Pt‐impy** exhibited a larger extinction coefficient in the ^1^ ^,3^MLCT absorption region than **Pt‐Me‐impy**, which is consistent with the theoretical MLCT calculation results (Table S4, Supporting Information). The energy gaps (E_g_) of 2.80 and 2.85 eV were obtained from the onset values of the UV–vis absorption band for **Pt‐impy** and **Pt‐Me‐impy**, respectively.

The photoluminescence (PL) emission spectra of the **Pt‐impy** and **Pt‐Me‐impy** were measured using a diluted MC solution at 298 K (RT) and a tetrahydrofuran (THF) solution at 77 K (Figure 3b; Figure S3a, Supporting Information). The peak wavelengths of **Pt‐impy** and **Pt‐Me‐impy** were 466 and 456 nm at RT, respectively. Compared with **BD‐02** containing a benzocarbene unit, **Pt‐impy** with a pyridocarbene unit exhibited a red‐shifted peak wavelength of 14 nm (Figure S5 and Table S3, Supporting Information). In contrast, **Pt‐Me‐impy** with a methyl conformation manager introduced into pyridocarbene exhibited only a wavelength shift of approximately 4 nm. The FWHM of the emission spectrum was 46 and 38 nm for **Pt‐impy** and **Pt‐Me‐impy**, respectively. Although the FWHM of the two Pt(II) complexes increased because of the improved MLCT characteristics compared to that of **BD‐02**, it was widened only by 2 nm in **Pt‐Me‐impy**. Therefore, the conformation manager effectively suppressed the strong red‐shift and minimized the broadening of the emission spectrum. Distinct vibrational peaks were observed in the PL spectra measured at 77 K (Figure 3b). The triplet energies of **Pt‐impy** and **Pt‐Me‐impy** were obtained as 2.75 and 2.79 eV, respectively, from the peak values of the corresponding spectra.

The oxidation potential was measured using cyclic voltammetry (CV), and the HOMO level of Pt(II) complexes was calculated from the onset of the oxidation curves (Figure S3b, Supporting Information). Since the HOMO is distributed across the phenyl‐O‐carbazole and Pt, the HOMO levels of **Pt‐impy** and **Pt‐Me‐impy** were similar, measured as −5.63 and −5.62 eV, respectively. The LUMO levels were calculated from the difference between the E_g_ and HOMO levels, yielding −2.83 and −2.77 eV for **Pt‐impy** and **Pt‐Me‐impy**, respectively. As predicted by the geometry optimization and time‐dependent density functional theory (TD‐DFT) results (Figures S1 and S4, Supporting Information), a shallower LUMO level was achieved in **Pt‐Me‐impy,** as the conformation manager increased the dihedral angle of the dtp unit and disrupted π‐conjugation extension. **Table** **1** summarizes the photophysical and electrochemical properties. Additionally, the electrochemical stability was measured using CV through 50 consecutive oxidation and 30 consecutive oxidation cycles under UV exposure (Figures S6 and S7, Supporting Information). Both **Pt‐impy** and **Pt‐Me‐impy** exhibited stable oxidation curves compared to **BD‐02**. Notably, **Pt‐impy** maintained its initial oxidation profile even after 50 consecutive cycles, indicating that the incorporation of the heteroatoms enhanced the oxidation stability and oxidation stability under the excitons.

**Table 1:** Photophysical, electrochemical properties, PLQY, exciton lifetimes, and photophysical parameters of **Pt‐impy** and **Pt‐Me‐impy**.

	UV–vis^a^ ^)^ [nm]	λ_RT/77 K_ ^b^ ^)^ [nm]	FWHM_RT/77 K_ ^b^ ^)^ [nm]	E_T1_ [eV]	E_g_ ^c^ ^)^ [eV]	HOMO/LUMO^d^ ^)^ [eV]	Φ_em_ ^e^ ^)^ [%]	τ^e^ ^)^ [µs]	Θ^f^ ^)^ [%]	*k*_r_ ^g^ ^)^ [× 10^5^s^−1^]	*k*_nr_ ^h^ ^)^ [× 10^4^s^−1^]
Pt‐impy	321;373	466/450	46/26	2.75	2.80	−5.63/−2.83	86	1.83	78	4.70	7.65
Pt‐Me‐impy	318;370	456/445	38/18	2.78	2.85	−5.62/−2.77	99	2.13	79	4.65	0.47

^a)^ MC solution (1.0 × 10^−5^ m).

^b)^ MC solution (1.0 × 10^−5^ m) at RT and THF solution (1.0 × 10^−5^ m) at 77 K.

^c)^ Optical energy gaps were estimated from the onset wavelengths of the UV–vis absorption spectra.

^d)^ HOMO levels were determined from the onset of the oxidation curves, whereas LUMO levels were calculated using the HOMO levels and E_g._

^e)^ 3‐CzPB:Pt dopant (40 nm, 95:5 wt.%) film.

^f)^ SiCzCz:SiTrzCz2:Pt dopant (40 nm, 30:5 wt.%) film.

^g)^ *k*_r_ = Φ_PL_/τ (radiative rate).

^h)^ *k*_nr_ = (1 − Φ_PL_)/τ (nonradiative rate).

To verify the potential of **Pt‐impy** and **Pt‐Me‐impy** as emitters for blue PhOLEDs, PLQY and time‐resolved PL were measured using 2,6‐bis(3‐(9*H*‐carbazol‐9‐yl)phenoxy)benzonitrile (3‐CzPB) films doped with Pt(II) complexes (Figure 3c and Table 1). In the PL spectra of the films, both **Pt‐impy** and **Pt‐Me‐impy** exhibited emission of only Pt(II) complexes due to energy transfer from the 3‐CzPB host. Although the emission peak was slightly red‐shifted due to the polarity of the host, the FWHM values of **Pt‐impy** and **Pt‐Me‐impy** were 43 and 31 nm, respectively. The FWHM of the **Pt‐Me‐impy** was slightly larger than that of the **BD‐02** (28 nm) in the doped film (Figures S8 and S9, Supporting Information). The PLQY values were 86% and 99% for **Pt‐impy** and **Pt‐Me‐impy**, respectively. The reduced nonradiative rate (*k*_nr_) due to the enhanced rigidity of the molecule caused by the methyl conformation manager and hydrogen bonding is responsible for the high PLQY of the **Pt‐Me‐impy** emitter. The exciton lifetimes (τ) were 1.83 and 2.13 µs for **Pt‐impy** and **Pt‐Me‐impy**, respectively, which were shorter than that of **BD‐02** (2.39 µs). The small FWHM, high PLQY, and short τ of **Pt‐Me‐impy** may allow high efficiency and long device lifetime in blue PhOLEDs.

### 2.3 Computational Analysis

To gain a deeper understanding of the molecular properties of **Pt‐impy** and **Pt‐Me‐impy**, density functional theory (DFT) and the TD‐DFT calculations were conducted using the optimally tuned ωB97xD (ω^*^B97xD) with LANL2DZ for Pt and Pople's triple zeta potential with double polarization functions (6‐311G**) for C, H, N, and O, as implemented in a suite of Gaussian 16 program.^[^ ^11^ ^]^

For the S_0_ state, the dihedral angle between 3,5‐di‐*tert*‐butylphenyl (dtp) and pyridocarbene in the **Pt‐impy** was calculated to be 41.12°, which was slightly increased to 54.82° in the **Pt‐Me‐impy** due to the steric hindrance caused by the methyl conformation manager (Figure S1a, Supporting Information). The corresponding dihedral angles for the T_1_ state were 32.80° and 49.95° for **Pt‐impy** and **Pt‐Me‐impy**, respectively, which follows the trend of the relative dihedral angle in the ground state. The changes in the dihedral angles between the S_0_ and T_1_ states were 8.32° and 4.87° for **Pt‐impy** and **Pt‐Me‐impy**, respectively. The small change in the dihedral angle in **Pt‐Me‐impy** suggests that the conformation manager plays a crucial role in enhancing the rigidity of the ligand during the transition between the S_0_ and T_1_ states. Additionally, the noncovalent interaction (NCI) calculations were conducted to identify the role of the conformation manager on the intramolecular interaction (Figure S1b, Supporting Information). The results show that the attractive intramolecular interaction between 3,5‐di‐*tert*‐butylphenyl (dtp) and pyridocarbene is strengthened by the introduction of the conformation manager due to the presence of multiple C–H…π interactions. This also confirms that the conformation manager plays a significant role in mitigating the energy loss process relevant to the structural change during the excitation and de‐excitation.

According to previous reports,^[^ ^12^ ^]^ the optimally tuned wB97xD (w^*^B97xD) functional guarantees a qualitative and quantitative result in predicting the T_1_ state of the phosphorescence materials. The optimal ω (ω*) values of **Pt‐impy** and **Pt‐Me‐impy** were set to 0.119 and 0.117 Bohr^−1^, respectively, which were determined from the minimum point of *J^2^* (ω) as a function of ω. The polarized continuum model (PCM) calculations without optimization were also conducted to obtain the excitation energies under the toluene medium. To explain the effect of the addition of the N heteroatom and CH_3_ unit on the LUMO level, the electron acceptability (ω^−^) values of the three carbene derivatives were calculated. As a result, the computed ω^−^values were 0.223, 0.351, and 0.312 eV, respectively, corresponding to the deeper LUMO energy level in the order of **Pt‐impy** > **Pt‐Me‐impy** > **BD‐02**. Table S4 (Supporting Information) presents the important photophysical parameters of Pt(II) complexes in excited state. The calculated T_1_ energies of **Pt‐impy** and **Pt‐Me‐impy** are 2.561 and 2.615 eV, respectively, which is consistent with the experimental results. The natural transition orbital calculations were also performed to assign the excitation characteristics of these complexes in the T_1_ state. As shown in Figure S10 (Supporting Information), the spatial distributions of the hole‐ and electron‐natural transition orbitals (HONTO and LUNTO) of **Pt‐impy** and **Pt‐Me‐impy** are very similar to those of **BD‐02**. The electron density of HONTO lies on the carbene, phenyl, Pt(II), and carbazole moieties, whereas that of LUNTO lies on the carbene, phenyl, Pt(II), di‐tertbutyl phenyl, and carbazole units. This result indicates that the effect of the substituted N heteroatom and methyl on the transition characteristic can be neglected. The excitation characteristics of **Pt‐impy** and **Pt‐Me‐impy** can be assigned as typical ^3^LE + ^3^MLCT transitions.

The ^3^MLCTs (%) of both Pt(II) complexes were calculated at the optimized T_1_ structures.^[^ ^13^ ^]^ Table S5 (Supporting Information) summarizes the electronic transitions and their corresponding contribution to ^3^MLCT. The calculated ^3^MLCT(%) of **Pt‐impy** and **Pt‐Me‐impy** are 9.42 and 9.24%, respectively, indicating that the **Pt‐impy** may show stronger ^3^MLCT character than **Pt‐Me‐impy_._** At the individual optimized T_1_ structures, the SOC constants between the S_0_ and T_1_ states (<T_1_|H_SOC_|S_0_>) were calculated using the M06/TZVP level of theory with the ZORA Hamiltonian using the ORCA 5.0 program.^[^ ^14^ ^]^ Interestingly, the <T_1_|H_SOC_|S_0_> of **Pt‐impy** and **Pt‐Me‐impy** were calculated to be 158.68 and 153.74 cm^−1^, which are slightly larger than that of **BD‐02** (Table S4, Supporting Information). Therefore, it is expected that **Pt‐impy** exhibits a faster *k*_r_ than **Pt‐Me‐impy**. With the same perspective, *k*_r_ of both materials is expected to be larger than that of **BD‐02**. According to the Einstein coefficient of spontaneous emission with first‐order perturbation theory, *k*_r_ can be obtained by means of the oscillation strength and excitation energy. However, the T_1_ level can be divided into three sublevels due to the SOC. Therefore, *k*_r_ of phosphorescence can be derived from the average of *k*_r_ of the three‐sublevels. The *k*_r_ of **BD‐02**, **Pt‐impy**, and **Pt‐Me‐impy** were calculated to be 9.97 × 10^5^, 1.23 × 10^6^, and 1.15 × 10^6^ s^−1^, respectively, which are qualitatively consistent with the experimental results. To indirectly understand the behavior of *k*_nr_ of **Pt‐impy** and **Pt‐Me‐impy**, the root mean square deviations (RMSDs) and total reorganization energies (*λ*_tot_) between the S_0_ and T_1_ states were calculated (Table S4, Supporting Information). The RMSD of **Pt‐impy** is 0.166, which is higher than that of **Pt‐Me‐impy** (0.150). Moreover, *λ*_tot_ values of **Pt‐impy** and **Pt‐Me‐impy** were calculated to be 2839.3 and 2754.1 cm^−1^ (Figure S11, Supporting Information). These results show that **Pt‐Me‐impy** exhibits a smaller *k*_nr_ than **Pt‐impy** due to the hindered structural changes associated with the energy loss during the S_0_→T_1_ and T_1_→S_0_ transitions. To clearly understand the role of methyl unit on reduction of *λ*_tot_, the representative vibration modes (*i*_th_) of **Pt‐impy** and **Pt‐Me‐impy** are assigned and shown in Figure S11b (Supporting Information). In **Pt‐impy**, the most contributable vibration modes to *λ*_tot_ can be assigned as 1075 cm^−1^ (*λ*_i_ = 63.1 cm^−1^), 1246 cm^−1^ (*λ*_i_ = 76.7 cm^−1^), 1293 cm^−1^(*λ*_i_ = 240.0 cm^−1^), and 1456 cm^−1^(*λ*_i_ = 37.0 cm^−1^), respectively. These correspond to vibration modes of 1072 cm^−1^ (*λ*_i_ = 20.1 cm^−1^), 1250 cm^−1^ (*λ*_i_ = 1.8 cm^−1^), 1286 cm^−1^ (*λ*_i_ = 197.6 cm^−1^), and 1457 cm^−1^ (*λ*_i_ = 15.7 cm^−1^) in **Pt‐Me‐impy**. From these results, it is worth noting that the calculated *λ*_i_ values of **Pt‐Me‐impy** are entirely smaller than those of **Pt‐impy**. This result indicates that methyl unit hinders the activation of specific vibration modes that significantly contribute to *λ*_tot_ during the excitation and de‐excitation. In other words, the presence of the methyl unit plays a pivotal role in suppressing the structural changes related to changes of vibration modes under the excitation and de‐excitation processes.

### 2.4 Device Performances

**Figure 4:**
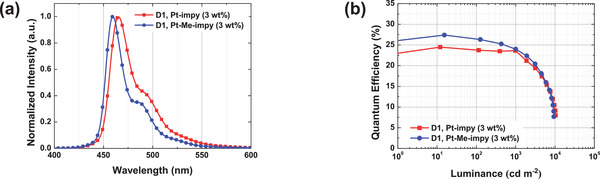
a) EL spectra and b) *L–*EQE curves of D1.

Multilayer blue PhOLEDs (**D1**) doped with **Pt‐impy** and **Pt‐Me‐impy** phosphors were fabricated via vacuum deposition using a 3‐CzPB host with a high triplet energy of 3.02 eV for the triplet exciton harvesting of Pt(II) complexes (Figure S12, Supporting Information).^[^ ^15^ ^]^ In the EL spectra of the PhOLEDs doped with 3 wt.% **Pt‐impy** and **Pt‐Me‐impy**, the emission peaks were observed at 465 and 460 nm, respectively (**Figure** **4**a and **Table** **2**). Pt‐Me‐impy exhibited a narrower FWHM of 21 nm compared to 27 nm for **Pt‐impy**, attributed to the methyl group acting as a conformation manager, which effectively suppressed the long‐wavelength broadening and the second vibrational peak. Therefore, **Pt‐Me‐impy** achieved a deep‐blue emission with a CIE_y_ coordinate of 0.109. In addition, the EQEs were 24.5% and 27.4% for **Pt‐impy** and **Pt‐Me‐impy** (Figure 4b), respectively, compared with 25.8% of the **BD‐02** device (Table 2; Table S6, Supporting Information). Considering that the PLQYs of **BD‐02, Pt‐impy**, and **Pt‐Me‐impy** are 95%, 86%, and 99%, respectively, the EQE is well correlated with the PLQY. The efficiency roll‐off in the Pt‐impy and Pt‐Me‐impy based PhOLEDs was relatively small. This can be explained by the short exciton lifetime, which helps suppressing exciton–exciton annihilation.

**Table 2:** Device performances of D1 and D2 for **Pt‐impy** and **Pt‐Me‐impy**.

Device (doping concentration)	V_d_ ^a^ ^)^ [V]	λ [nm]	FWHM [nm]	CIE		EQE (%)		Roll‐off^b^ ^)^ [%]	LT_95_ [h]
				x	y	[1000 cd m^−2^]	[max]		
D1, Pt‐impy (3 wt.%)	5.0	465	27	0.130	0.161	23.6	24.5	4	–
D1, Pt‐Me‐impy (3 wt.%)	5.0	460	21	0.134	0.109	24.0	27.4	12	–
D2, Pt‐impy (8 wt.%)	4.6	472	40	0.133	0.245	19.2	20.5	6	431
D2, Pt‐Me‐impy (8 wt.%)	4.4	465	26	0.136	0.173	20.5	22.1	7	136
D2, Pt‐impy (12 wt.%)	4.8	473	42	0.135	0.263	17.9	18.7	4	543
D2, Pt‐Me‐impy (12 wt.%)	4.4	466	27	0.136	0.180	20.3	22.8	11	228

^a)^ Driving voltage at 1 000 cd m^−2^.

^b)^ (EQE_max_‐EQE_1000nit_)/EQE_max_ × 100.

**Figure 5:**
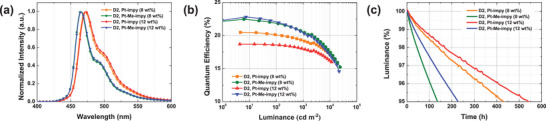
a) EL spectra, b) *L–*EQE curves, and c) device lifetime of D2 at the initial luminance of 1000 cd m^−2^.

**Figure 6:**
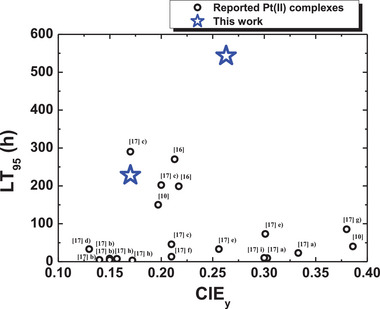
Comparison of the device performance of the reported Pt(II) complexes.^[^ ^10^, ^16^, ^17^ ^]^

The device performances of **the Pt‐impy** and **Pt‐Me‐impy** PhOLEDs were further confirmed by optimizing the device structure (**D2**) using the architecture described in Figure S13 (Supporting Information). Deuterated hosts were used in this study for enhanced operational stability based on previous reports.^[^ ^16^ ^]^ At a doping concentration of 8 wt.%, the **Pt‐Me‐impy** device exhibited an EQE of 22.1%, a color coordinate of (0.136, 0.173), and an LT95 of 136 h. When the doping concentration was increased to 12 wt.%, the performance was further improved, reaching an EQE of 22.8%, a color coordinate of (0.136, 0.180), and a prolonged LT95 of 228 h. In the case of **Pt‐impy** devices, they exhibited an EQE of 20.5%, a color coordinate of (0.133, 0.245), and a notably longer LT95 of 431 h at 8 wt.%. Upon increasing the doping level to 12 wt.%, the LT95 of **Pt‐impy** was further extended to 543 h (**Figure** **5** and Table 2). The device lifetime of **Pt‐Me‐impy** is one of the best lifetimes ever reported in the blue PhOLEDs with CIE_y_ of less than 0.20. Moreover, the LT_95_ of the **Pt‐impy** device is the longest device lifetime achieved among all blue PhOLEDs. Considering that the LT_95_ of the **BD‐02** device is only 150 h with color coordinate (0.141, 0.197) in the previous work,^[^ ^10^ ^]^ and LT_95_ of 88 h with a color coordinate of (0.140, 0.170) in the **D2** device structure (Figure S14 and Table S7, Supporting Information), the **Pt‐impy** and **Pt‐Me‐impy** achieved remarkably improved device lifetime in the blue PhOLEDs. The introduction of the pyridocarbene unit extended the device lifetime of the **Pt‐impy** and **Pt‐Me‐impy** by a short triplet exciton lifetime, intensified ^3^MLCT character, strong SOC, and resistance to electrochemical oxidation. Moreover, the methyl conformation manager narrowed the emission spectrum for high color purity in the **Pt‐Me‐impy** blue PhOLEDs. The shortened triplet exciton lifetime closely related to the ^3^MLCT characteristics and SOC relieves the stress of the phosphor during the emission process, assisting the stable operation of the phosphor. As demonstrated in the repetitive oxidation cycles in the electrochemical measurement, the stability against oxidation is critical to the long device lifetime. This is because the holes are trapped by the phosphor in the emitting layer, as can be predicted from the energy levels of the hosts and phosphor. The device performances of the **Pt‐impy** and **Pt‐Me‐impy** were compared with those of other blue phosphors reported in the literature (**Figure** **6**).

## 3 Conclusion

In this study, two Pt(II) complexes, **Pt‐impy** and **Pt‐Me‐impy**, were developed by replacing the benzimidazole of the tetradentate Pt(II) complexes with imidazo[4,5‐*b*]pyridine, which deepened the LUMO and intensified ^3^MLCT characteristics. The strong ^3^MLCT characteristics increased the SOC and largely shortened the exciton lifetime, resulting in triplet exciton lifetimes of 1.83 and 2.13 µs for **Pt‐impy** and **Pt‐Me‐impy**, respectively, compared to 2.39 µs of **BD‐02** phosphor. Despite the strong ^3^MLCT characteristics, the **Pt‐Me‐impy** with a methyl conformation manager embedded imidazo[4,5‐*b*]pyridine achieved a small FWHM of 21 nm, color coordinate of (0.134, 0.109), and high EQE of 27.4%. In particular, the optimized **Pt‐Me‐impy** blue PhOLED demonstrated a LT_95_ device lifetime of 228 h at 1000 cd m^−2^. The **Pt‐impy** device achieved LT_95_ of 543 h at 1000 cd m^−2^, which is one of the best device lifetimes reported in the blue PhOLEDs. Therefore, the design strategy employing pyridocarbene and an additional methyl conformation manager in the ligand of the tetradentate Pt(II) complexes is effective in achieving a long device lifetime in the blue PhOLEDs.

## Conflict of Interest

The authors declare no conflict of interest.

## Supporting information



Supporting Information

## Data Availability

The data that support the findings of this study are available in the supplementary material of this article.
