# Supplementary material for: Pyridocarbene‐Based Tetradentate Pt(II) Complexes for Long Device Lifetime over 500 h in Blue Phosphorescent Organic Light–Emitting Diodes
Source: Adv Mater. 2025 Aug 19;37(44):e10070. doi: 10.1002/adma.202510070 (PMC12592909; doi:10.1002/adma.202510070)
Supplement: Supplementary file 1 — Supporting Information [file ADMA-37-e10070-s001.doc]

Supporting Information

Pyridocarbene Based Tetradentate Pt(II) Complexes for Long Device Lifetime Over 500 h in Blue Phosphorescent Organic Light-Emitting Diodes

Kiun Cheong^1+^, Hyunjung Lee^1+^, Jangho Moon^2^, Chan Hee Ryu^3^, Gyeong Woo Kim^4^, Jun Yun Kim^4^, In-Ho Lee^5^, Yong-Woo Kim^6^, Young Woong Lee^6^, Seokhyeon Yu^6^, Kang Mun Lee^3^*, Sunwoo Kang^7^*, Jun Yeob Lee^1,2,8^*

^1^School of Chemical Engineering, Sungkyunkwan University 2066, Seobu-ro, Jangan-gu, Suwon-si, Gyeonggi, 16419, Republic of Korea

^2^Department of Display Convergence Engineering, Sungkyunkwan University 2066, Seobu-ro, Jangan-gu, Suwon, Gyeonggi, 16419, Republic of Korea

^3^Department of Chemistry, Institute for Molecular Science and Fusion Technology, Kangwon National University, Chuncheon, Gangwon, 24341, Republic of Korea.

^4^LG Display Co. Ltd., LG Science Park, 30, Magokjungang 10-ro, Gangseo-gu, Seoul, 07796, Republic of Korea

^5^P&H Tech, 16-25, Dongbaekjungang-ro 16beon-gil, Giheung-gu, Yongin-si, Gyeonggi, 17015, Republic of Korea

^6^LT Materials, 113-19, Dangha-ro, Namsa-eup, Cheoin-gu, Yongin-si, Gyeonggi 17118, Republic of Korea

^7^Department of Chemistry, Dankook University, Cheonan, Chungnam, 31116, Republic of Korea

^8^SKKU Institute of Energy Science and Technology, Sungkyunkwan University 2066, Seobu-ro, Jangan-gu, Suwon, Gyeonggi, 16419, Republic of Korea

^+^ Kiun Cheong and Hyunjung Lee contributed equally to this work

* Corresponding author

E-mail: [kangmunlee@kangwon.ac.kr](mailto:kangmunlee@kangwon.ac.kr), sunwoo.kang@dankook.ac.kr, [leej17@skku.edu](mailto:leej17@skku.edu)

**Table of Contents**

**1. General information**

**2. Computational details**

**3. X-ray crystallography**

**4. Device fabrication**

**5. Experimental Procedures**

**6. Supplementary Figures**

**7. Supplementary Tables**

**8. ^1^H and ^13^C NMR**

**9. Reference**

**1. General information**

All chemical compounds were commercially available. Reaction reagents were purchased from Sigma Aldrich Co., Alfa aesar Co., and TCI Co. Palladium catalysts were purchased from P&H Tech Co. Solvents were purchased from Samchun Pure Chemical CO., Ltd., Duksan Sci. Co., and Daejung Chemical & Metal Co. ^1^H and ^13^C nuclear magnetic resonance (NMR) spectra were measured on Unity Inova 500 MHz spectrometer. Chloroform-*d*_3_ (CDCl_3_) and methylene chloride-*d*_2_ (CD_2_Cl_2_) were used for NMR analysis. UV-vis spectrophotometer (JASCO, V-730) and fluorescence spectrometer (PerkinElmer, LS-55) were used for UV-vis spectra and PL spectra, respectively. The HOMO levels were estimated using a cyclic voltammetry (CV) (Ivium Tech., Iviumstat). CV measurement was carried out in dichloromethane solution with scan rate at 100 mV/s. The platinum wires were used as working and counter and Ag/AgCl was used as reference electrode respectively. Internal standard was ferrocenium/ferrocene couple and supporting electrolyte was 0.1 M tetrabutylammonium perchlorate (TBAClO_4_). The mass spectra were measured using a JMS-700 (JEOL) with high resolution fast atom bombardment (FAB) mode and Advion Expression-L CMS spectrometer in APCI mode. PL quantum yield and transient PL decay data were obtained using Quantaurus QY Absolute system (Hamamatsu, C11347-11) and Hamamatsu Quantaurus-Tau system (Hamamatsu, C11367-31).

**2. Computational details**

Density functional theory (DFT) calculations and time-dependent DFT (TD-DFT) were performed using the Gaussian 16 program package.^[1]^ The ground state geometries were optimized using B3LYP functional and LANL2DL basis set for Pt and B3LYP^[2]^/6-31g(d,p) for light elements (C, H, N and O). The computations of the singlet and triplet transition energies were carried out using TD-DFT by same method based on the optimized ground state geometry.

**3. X-ray crystallography**

Single X-ray quality **Pt-Me-impy** crystals were grown from mixtures of DCM and *n-*hexane. A single crystal was coated with Paratone oil and mounted on a glass capillary. Crystallographic measurements were performed using a Bruker D8QUEST CCD area detector diffractometer with graphite monochromated Mo Kα radiation (*λ* = 0.71073 Å). The structure for **Pt-Me-impy** was assessed using direct methods, and all nonhydrogen atoms were subjected to anisotropic refinement with the full-matrix least-squares method on *F*2 using a SHELXTL/PC software package. The **Pt-Me-impy** Xray crystallographic data are available in CIF format (CCDC‒2427330) and are provided free of charge by The Cambridge Crystallographic Data Centre. Hydrogen atoms were placed at their geometrically calculated positions and refined using a riding model on the corresponding carbon atoms with isotropic thermal parameters. The detailed crystallographic data are given in **Tables S1** and **S2**.

**4. Device fabrication**

Vacuum thermal evaporation was used for device fabrication. The encapsulated devices were maintained at ambient conditions, and their electrical and optical properties were evaluated using Keithley 2400 and Konica Minolta CS-2000 source meters, respectively.

The device structure of D1 is as follows: indium tin oxide (ITO, 50 nm)/PEDOT:PSS (40 nm)/TAPC (10 nm)/TCTA (5 nm)/mCP (5 nm)/3-CzPB:Pt emitters (25 nm: 3 wt%)/TSPO1 (25 nm)/LiF (1.5 nm)/Al (200 nm), where PEDOT:PSS is poly(3,4-ethylenedioxythiophene) polystyrene sulfonate, TAPC is 1,1-bis[(di-4-tolylamino)phenyl]cyclohexane, TCTA is tris(4-carbazoyl-9-ylphenyl)amine, mCP is 3-di(9*H*-carbazol-9-yl)benzene, and TSPO1 is diphenyl[4-(triphenylsilyl)phenyl]phosphine oxide. **Figure S12** shows the molecular structures of the materials used to fabricate the PhOLEDs.

The device structure of D2 is as follows: ITO (50 nm)/BCFN:NDP-9 (10nm, 5%)/BCFN (25 nm)/BPCzCz (5 nm)/d-BPCzCz:d-SiTrzCz2:Pt dopant (50 nm, 46:46:8 wt% and 44:44:12 wt%)/SiTrzCz2 (5 nm)/mSiTrz:Liq (20 nm)/LiF (1 nm)/Al (90 nm), where BFCN is *N*-([1,1′-biphenyl]-4-yl)-9,9-dimethyl-*N*-(4-(9-phenyl-9*H*-carbazol-3-yl)phenyl)-9*H*-fluoren-2-amine, NDP-9 is 2-(7-dicyanomethylene-1,3,4,5,6,8,9,10-octafluoro-7*H*-pyren-2-ylidene)-malononitrile, BPCzCz is 9-([1,1'-biphenyl]-3-yl)-9*H*-3,9'-bicarbazole, SiTrzCz2 is 9,9'-(6-(3-(triphenylsilyl)phenyl)-1,3,5-triazine-2,4-diyl)bis(9*H*-carbazole), mSiTrz is 2-phenyl-4,6-bis(3-(triphenylsilyl)phenyl)-1,3,5-triazine. Details of the devices are mentioned in the **Figure S13**.

**5. Experimental Procedures**

**3-((9-(4-(*tert*-butyl)pyridin-2-yl)-9*H*-carbazol-2-yl)oxy)aniline (L3)**

9-(4-methylpyridin-2-yl)-9*H*-carbazol-2-ol (**L4**) (18.0 g, 569 mmol)^[3]^, 3-bromoaniline (7.4 mL, 68.2 mmol), CuI (3.2 g, 17.0 mmol), 2-Picolinic acid (11.2 g, 90.8 mmol) and K_3_PO_4_ (48 g, 227.2 mmol) were added and dissolved in DMSO 100 mL into a two-neck flask. The flask was stirred at 100 °C for 12 h. After that, the product was extracted using EA/water and washed by brine. A brown powder was obtained after further purification by column chromatography using an ethyl acetate (EA):methylene chloride (MC) (1:30) eluent (12.5 g, yield 55%). ^1^H NMR (500 MHz, DMSO-*d*_6_) δ 8.60 (d, *J* = 5.3 Hz, 1H), 8.23 (d, *J* = 8.4 Hz, 1H), 8.20 (d, *J* = 7.6 Hz, 1H), 7.77 (dt, *J* = 8.3, 0.9 Hz, 1H), 7.62 (d, *J* = 1.8 Hz, 1H), 7.47 (dd, *J* = 5.3, 1.7 Hz, 1H), 7.43 (ddd, *J* = 8.3, 7.1, 1.3 Hz, 1H), 7.33 (td, *J* = 7.5, 1.0 Hz, 1H), 7.26 (d, *J* = 2.1 Hz, 1H), 7.04 (dd, *J* = 8.4, 2.1 Hz, 1H), 7.00 (t, *J* = 7.9 Hz, 1H), 6.33 (ddd, *J* = 8.1, 2.1, 0.9 Hz, 1H), 6.24 (t, *J* = 2.2 Hz, 1H), 6.21 (ddd, *J* = 7.9, 2.4, 0.9 Hz, 1H), 5.20 (s, 2H), 1.33 (s, 9H). MS (APCI) m/z 409.5 [(M+H)^+^].

***N*-(3-((9-(4-(*tert*-butyl)pyridin-2-yl)-9*H*-carbazol-2-yl)oxy)phenyl)-3-nitropyridin-2-amine (L2-H)**

3-((9-(4-(*tert*-butyl)pyridin-2-yl)-9*H*-carbazol-2-yl)oxy)aniline (**L3**) (6.0 g, 15 mmol), 2-chloro-3-nitropyridine (7.6 g, 37 mmol), Pd_2_(dba)_3_ (0.68 g, 0.75 mmol), di-tert-butyl(2,2-diphenyl-1-methyl-1-cyclopropyl)phosphine (cBRIDP) (0.79 g, 2.2 mmol), and NaO*t*Bu (2.8 g, 30 mmol) were added and dissolved in toluene (60 mL) into a two-neck flask. The flask was stirred at 110 °C for 12 h. After that, the product was extracted using MC/water and washed by brine. An orange powder was obtained after further purification by column chromatography using a methylene chloride (MC):hexane (1:4) eluent (7.9 g, yield 98%). ^1^H NMR (500 MHz, CD_3_Cl): δ 10.11 (s, 1H), 8.58 (d, J = 5.4 Hz, 1H), 8.40 (dd, J = 4.3, 1.4 Hz, 1H), 8.06 (d, J = 8.4 Hz, 2H), 7.77 (d, J = 8.3 Hz, 1H), 7.56 (d, J = 1.2 Hz, 1H), 7.49 (d, J = 1.9 Hz, 1H), 7.45 – 7.39 (m, 2H), 7.34 – 7.27 (m, 4H), 7.08 (dd, J = 8.4, 2.1 Hz, 1H), 6.85 (ddd, J = 7.8, 2.2, 1.4 Hz, 1H), 6.82 – 6.77 (m, 1H), 1.34 (s, 9H). MS (APCI) m/z 530.2 [(M+H)^+^].

**2-(3-(3*H*-imidazo[4,5-*b*]pyridin-3-yl)phenoxy)-9-(4-(*tert*-butyl)pyridin-2-yl)-9*H*-carbazole (L1-H)**

**L2-H** (3.4 g, 5.7 mmol), formic acid (160 mL), Fe powder (8.9 g, 159 mmol), and ammonium chloride (8.5 g, 159 mmol) were added and dissolved in 2-propanol (150 mL) into a two-neck flask. The flask was stirred at 110 °C for 12 h. After that, the product was filtered by using a short silica column with MC. The filtrate was extracted using MC/water and washed by brine. A pale yellow powder was obtained after further purification by column chromatography using an ethyl acetate (EA):hexane (1:4) eluent (3.8 g, yield 49%) ^1^H NMR (500 MHz, CD_3_Cl): δ 8.58 (d, J = 5.3 Hz, 1H), 8.46 (dd, J = 4.8, 1.4 Hz, 1H), 8.41 (s, 1H), 8.17 (dd, J = 8.1, 1.4 Hz, 1H), 8.09 (t, J = 7.2 Hz, 2H), 7.74 (d, J = 8.3 Hz, 1H), 7.58 (d, J = 1.9 Hz, 2H), 7.52 – 7.47 (m, 3H), 7.45 – 7.40 (m, 1H), 7.34 – 7.30 (m, 2H), 7.29 (dd, J = 5.3, 1.7 Hz, 1H), 7.12 – 7.09 (m, 2H), 1.35 (s, 9H). MS (APCI) m/z 510.2 [(M+H)^+^].

**3-(3-((9-(4-(*tert*-butyl)pyridin-2-yl)-9*H*-carbazol-2-yl)oxy)phenyl)-1-(3,5-di-*tert*-butylphenyl)-3*H*-imidazo[4,5-*b*]pyridin-1-ium trifluoromethanesulfonate (L-H)**

**L1-H** (3.8 g, 5.3 mmol), (3,5-di-*tert*-butylphenyl)(mesityl)iodonium trifluoromethane sulfonate^[4]^ (6.5 g, 11.1 mmol) and copper acetate (80.5 mg, 0.44 mmol) were added into a two-neck flask and dissolved in dimethylformamide (37 mL). The flask was stirred at 130 °C at 12 h. After that, a product was filtered roughly through a short pad of silica and washed with EA:MC (1/9) eluent. Brown powder was obtained without further purification and used it in next metalation (4.5 g, yield 72%).

**Platinum(II) 1-(3-((9-(4-(*tert*-butyl)pyridin-2-yl*-κ*N)-9*H*-carbazol-2-yl-*κ*C^1^)oxy)phenyl*-κ*C^1^)-3-(3,5-di-*tert*-butylphenyl)-3*H*-imidazo[4,5-*b*]pyridin-2-ylidene-*κ*C^2^ (Pt-impy)**

**L-H** (4.5 g, 5.3 mmol), dichloro(1,5-cyclooctadiene)platinum(II) (Pt(cod)Cl_2_) (2.0 g, 5.3 mmol), and sodium acetate (NaOAc) (1.3 g, 15.9 mmol) were added it into a two-neck flask and dissolved in DMF (100 mL). The flask was stirred at 160 °C for 12 h. After that, the reaction was extracted using MC and water. A yellow powder was obtained after further purification by column chromatography using an MC:hexane (1:1) eluent (1.8 mg, yield 27%, purity 99.6%). ^1^H NMR (500 MHz, CD_2_Cl_2_): δ 8.68 (d, J = 6.2 Hz, 1H), 8.59 (dd, J = 4.8, 1.4 Hz, 1H), 8.34 (dd, J = 6.3, 2.1 Hz, 1H), 8.03 – 7.97 (m, 1H), 7.81 (d, J = 8.2 Hz, 1H), 7.67 – 7.61 (m, 2H), 7.59 (s, 1H), 7.51 (s, 2H), 7.37 (d, J = 8.2 Hz, 1H), 7.36 – 7.25 (m, 4H), 7.08 – 6.99 (m, 2H), 5.58 (d, J = 4.8 Hz, 1H), 1.24 (br, s, 18H), 0.87 (s, 9H). ^13^C NMR (125MHz, CD_2_Cl_2_) δ 192.8, 161.7, 156.0, 154.0, 152.5, 151.8, 149.0, 147.6, 146.1, 145.0, 143.6, 138.7, 136.0, 128.9, 128.8, 124.1, 123.6, 122.8, 122.2, 119.7, 119.1, 118.5, 116.7, 116.2, 115.4, 115.3, 114.1, 113.4, 112.5, 111.4, 110.2, 110.0, 34.8, 31.2, 29.5. HRMS (FAB+) m/z 891.3348 [(M+H)^+^]. Calculated for C_47_H_45_N_5_OPt: 890.3272

***N*-(3-((9-(4-(*tert*-butyl)pyridin-2-yl)-9*H*-carbazol-2-yl)oxy)phenyl)-4-methyl-3-nitropyridin-2-amine (L2-Me)**

**L2-Me** was synthesized by using the same method for synthesis of **L2-H**. 2-chloro-4-methyl-3-nitropyridine (6.4 g, 37.3 mmol) was used and a product was obtained after further purification by column chromatography using an EA/hexane (1:9) eluent (5.3 g, yield 66%). ^1^H NMR (500 MHz, CD_3_Cl): δ 9.11 (s, 1H), 8.57 (d, J = 5.3 Hz, 1H), 8.06 (d, J = 7.6 Hz, 1H), 8.06 (d, J = 8.4 Hz, 1H), 7.76 (d, J = 8.2 Hz, 1H), 7.55 (d, J = 1.4 Hz, 1H), 7.47 (d, J = 2.1 Hz, 1H), 7.44 (t, J = 2.2 Hz, 1H), 7.43 – 7.39 (m, 1H), 7.30 (dd, J = 16.0, 8.7 Hz, 2H), 7.27 (d, J = 5.8 Hz, 1H), 7.18 (dd, J = 8.1, 1.2 Hz, 1H), 7.06 (dd, J = 8.4, 2.1 Hz, 1H), 6.80 (dd, J = 7.8, 2.0 Hz, 1H), 6.65 (d, J = 4.9 Hz, 1H), 2.54 (s, 3H), 1.34 (s, 9H). MS (APCI) m/z 544.2 [(M+H)^+^].

**9-(4-(*tert*-butyl)pyridin-2-yl)-2-(3-(7-methyl-3*H*-imidazo[4,5-*b*]pyridin-3-yl)phenoxy)-9*H*-carbazole (L1-Me)**

**L1-Me** was synthesized by using the same method for synthesis of **L1-H**. **L2-Me** (5.3 g, 9.8 mmol) was used and a product was obtained after further purification by column chromatography using an EA/hexane (1:1) eluent (3.9 g, yield 76%). ^1^H NMR (500 MHz, CD_3_Cl): δ 8.59 (d, J = 5.2 Hz, 1H), 8.39 (d, J = 4.9 Hz, 1H), 8.10 (t, J = 7.9 Hz, 2H), 7.75 – 7.69 (m, 1H), 7.59 (d, J = 1.9 Hz, 2H), 7.52 (t, J = 8.1 Hz, 1H), 7.47 (d, J = 1.0 Hz, 1H), 7.46 – 7.41 (m, 2H), 7.35 – 7.32 (m, 1H), 7.31 – 7.29 (m, 1H), 7.22 (d, J = 4.9 Hz, 1H), 7.15 (dd, J = 8.2, 1.5 Hz, 1H), 7.09 (dd, J = 8.4, 2.1 Hz, 1H), 2.81 (s, 3H), 1.36 (s, 9H). MS (APCI) m/z 524.2 [(M+H)^+^].

**3-(3-((9-(4-(*tert*-butyl)pyridin-2-yl)-9*H*-carbazol-2-yl)oxy)phenyl)-1-(3,5-di-*tert*-butylphenyl)-7-methyl-3*H*-imidazo[4,5-*b*]pyridin-1-ium trifluoromethanesulfonate (Ph-L)**

**L-Me** was synthesized by using the same method for synthesis of **L-H**. **L1-Me** (1.6 g, 3.1 mmol) was used and a product was filtered roughly through a short pad of silica and washed with acetone:MC (1:10) eluent. Brown powder was obtained without further purification and used it in next metalation (2.2 g, yield 82%).

**Platinum(II) 1-(3-((9-(4-(*tert*-butyl)pyridin-2-yl*-κ*N)-9*H*-carbazol-2-yl-*κ*C^1^)oxy)phenyl*-κ*C^1^)-3-(3,5-di-*tert*-butylphenyl)-4-methyl-3*H*-imidazo[4,5-*b*]pyridin-2-ylidene-*κ*C^2^ (Pt-Me-impy)**

**Pt-Me-impy** was synthesized by using the same method for synthesis of **Pt-impy**. **L-Me** (2.0 g, 2.5 mmol) was used and a product was obtained after further purification by column chromatography using an MC:hexane (1:1) eluent (0.86 mg, yield 38%, purity 99.6%). ^1^H NMR (500 MHz, CD_2_Cl_2_): δ 8.60 (d, J = 6.2 Hz, 1H), 8.44 (dd, J = 7.3, 1.3 Hz, 1H), 8.40 (d, J = 4.9 Hz, 1H), 8.04 – 7.97 (m, 1H), 7.78 (d, J = 8.2 Hz, 1H), 7.63 (d, J = 7.7 Hz, 1H), 7.61 (d, J = 1.7 Hz, 1H), 7.48 (s, 2H), 7.36 – 7.27 (m, 4H), 7.09 – 6.99 (m, 3H), 5.73 (dd, J = 6.2, 1.7 Hz, 1H), 1.94 (s, 3H), 1.50 (br, s, 9H), 1.06 (br, s, 9H), 0.94 (s, 9H). ^13^C NMR (125MHz, CD_2_Cl_2_) δ 191.8, 161.7, 155.6, 154.2, 152.1, 149.3, 147.9, 145.9, 144.4, 143.9, 138.7, 137.3, 132.3, 128.8, 127.9, 124.0, 123.7, 123.1, 122.2, 121.8, 119.8, 116.2, 116.1, 115.5, 115.4, 114.1, 113.3, 112.5, 111.6, 110.8, 110.5, 34.9, 31.2, 29.6, 18.5. HRMS (FAB+) m/z 905.3504 [(M+H)^+^]. Calculated for C_48_H_47_N_5_OPt: 904.3429

**6. Supplementary Figures**

**
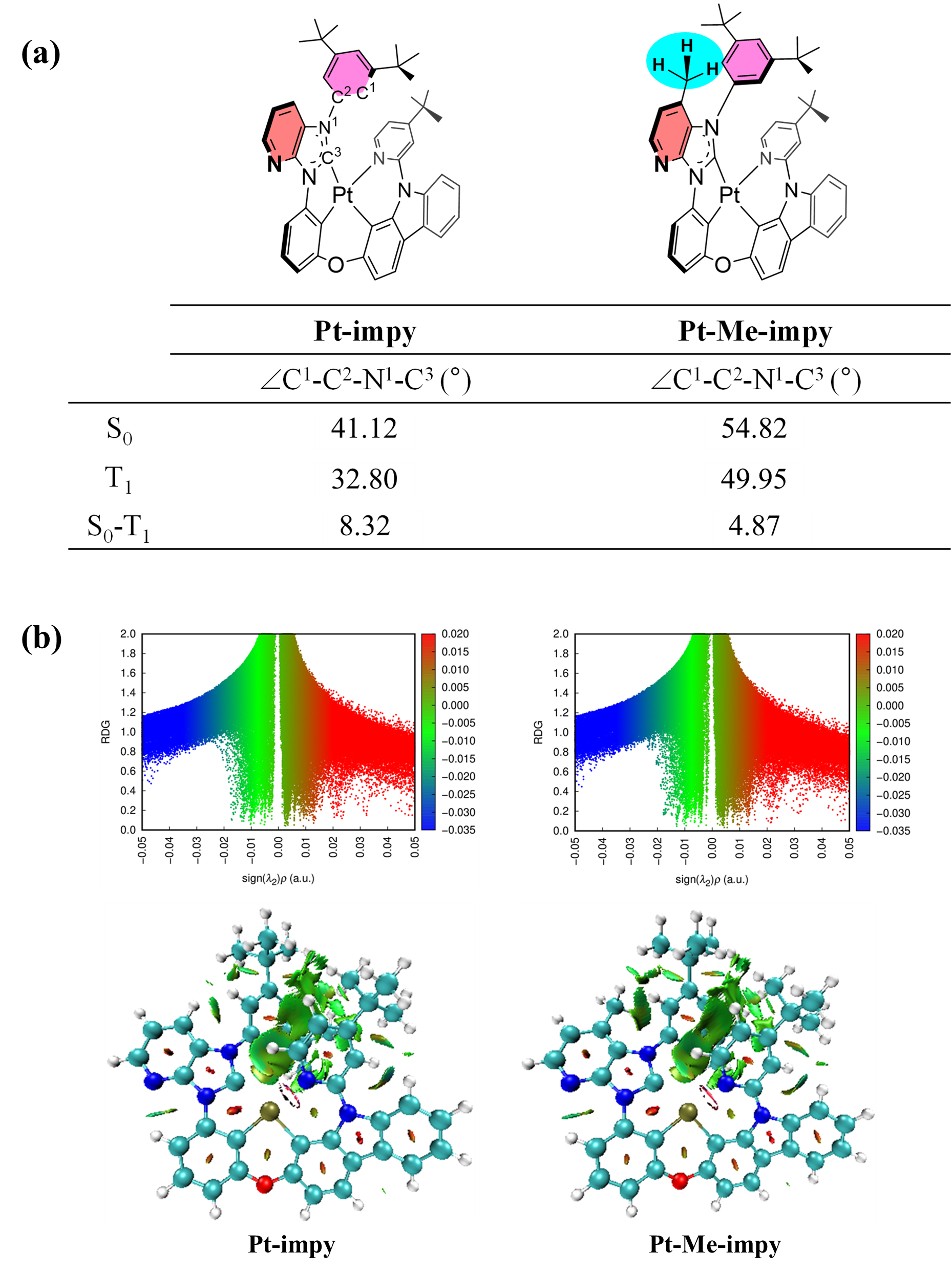
**

**Figure S1.** (a) Dihedral angles of the **Pt-impy** and **Pt-Me-impy** in S_0_ and T_1_ states. (b) The NCI plots of the **Pt-impy** and **Pt-Me-impy** in the S_0_ state.

**
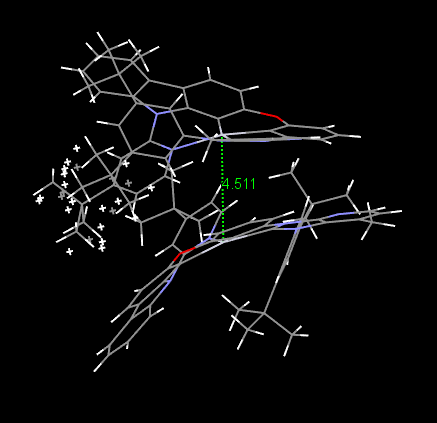
**

**Figure S2**. X-ray crystal structure of **Pt-Me-impy**.

**Figure S3**. (a) PL spectra at RT and (b) CV curve of **Pt-impy** and **Pt-Me-impy**.

**Figure S4**. HOMO and LUMO distributions of the **Pt-impy** and **Pt-Me-impy**.

**Figure S5**. (a) UV-vis absorption, PL spectra at RT and 77K and (b) CV curve of **BD-02**.

**Figure S6**. Oxidation CV curves of (a) **BD-02**, (b) **Pt-impy**, and (c) **Pt-Me-impy** at 50 cycles for electrochemical stability measurements.

**Figure S7**. Oxidation CV curves of (a) **BD-02**, (b) **Pt-impy**, and (c) **Pt-Me-impy** at 30 cycles for electrochemical stability measurements under UV exposure.

**Figure S8**. PL spectra of **Pt-impy** and **Pt-Me-impy** with 3-CzPB host.

**Figure S9**. (a) PL spectrum, (b) TRPL curves of **BD-02** doped with 3-CzPB host.

**Figure S10**. The natural transition orbitals of **BD-02**, **Pt-impy**, and **Pt-Me-impy** in the T_1_ state.


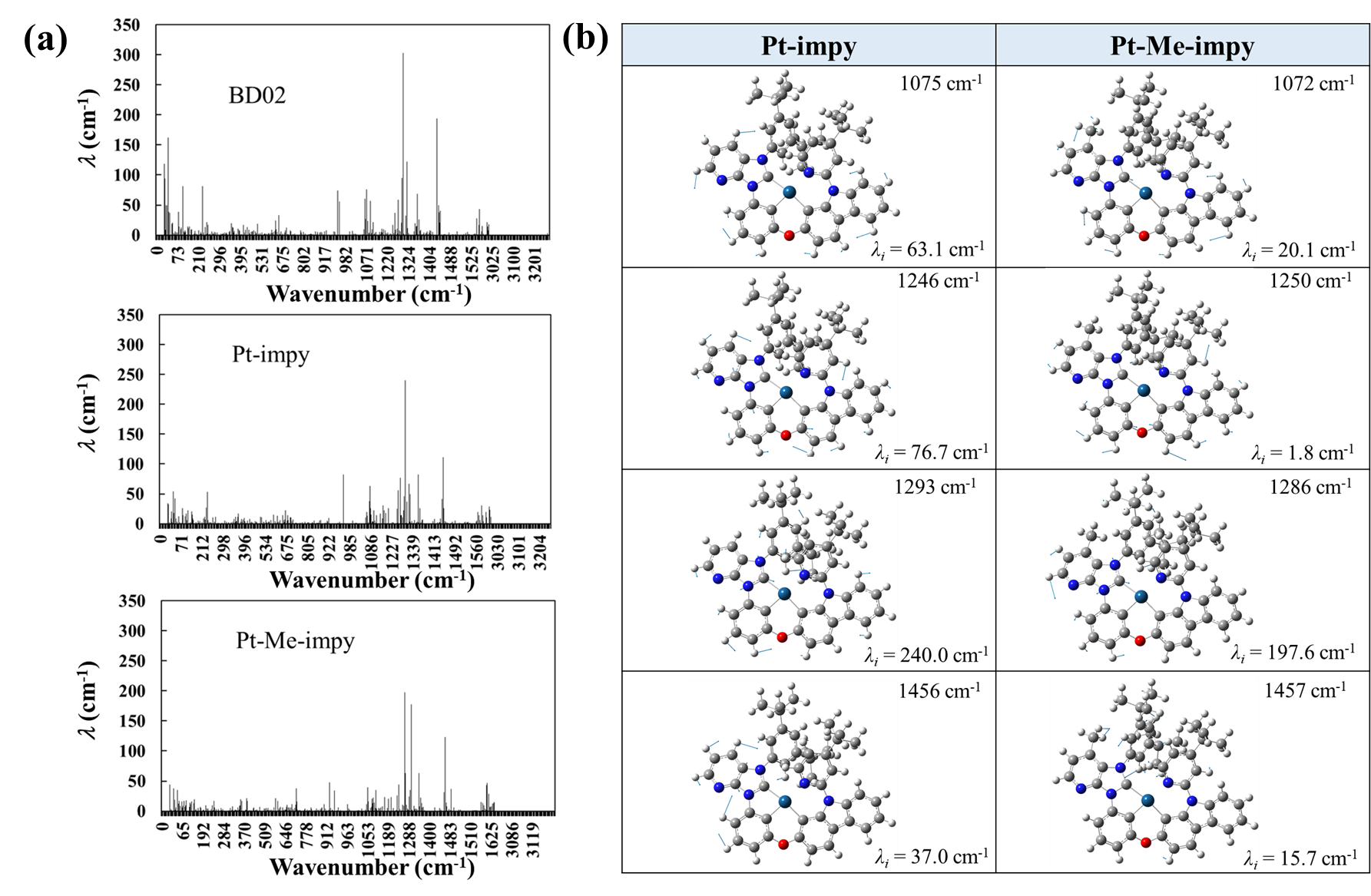


**Figure S11**. (a) The reorganization energy of **BD-02**, **Pt-impy**, and **Pt-Me-impy** as a function of vibration wavenumber between S_0_ and T_1_ states. (b) The representative vibration modes contributing to reorganization energy of **Pt-impy** and **Pt-Me-impy**.

**Figure S12**. (a) Energy diagram of D1 with **Pt-impy** and **Pt-Me-impy**. (b) The molecular structure of materials used for fabricating D1.

**Figure S13**. (a) Energy diagram of D2 with **Pt-impy** and **Pt-Me-impy**. (b) The molecular structure of materials used for fabricating D2.

**Figure S14**. (a) EL spectrum, (b) *L–*EQE curves and (c) device lifetime of D2 device of BD-02 at an initial luminance of 1000 cd m^−2^.

**7. Supplementary Tables**

**Table S1**. Crystal data and structure refinement for **Pt-Me-impy**.

Empirical formula C_48_ H_47_ N_5_ O Pt

Formula weight 904.99

Temperature 296(2) K

Wavelength 0.71073 Å

Crystal system Triclinic

Space group *P*-1

Unit cell dimensions a = 14.678(2) Å α = 75.635(4)°

b = 17.154(3) Å β = 88.575(4)°

c = 19.084(3) Å γ = 69.875(4)°

Volume 4361.2(11) Å^3^

Z 4

Density (calculated) 1.378 Mg/m^3^

Absorption coefficient 3.257 mm^-1^

F(000) 1824

Crystal size 0.152 x 0.034 x 0.032 mm^3^

Theta range for data collection 2.509 to 28.293°.

Index ranges –19<=h<=19, –22<=k<=22, –25<=l<=25

Reflections collected 130698

Independent reflections 21019 [R(int) = 0.0881]

Completeness to theta = 25.242° 99.8 %

Absorption correction Semi-empirical from equivalents

Max. and min. transmission 0.7456 and 0.5722

Refinement method Full-matrix least-squares on F^2^

Data / restraints / parameters 21019 / 252 / 1082

Goodness-of-fit on F^2^ 1.037

Final R indices [I>2sigma(I)] R1 = 0.0424, wR2 = 0.0760

R indices (all data) R1 = 0.0763, wR2 = 0.0882

Largest diff. peak and hole 0.535 and –0.524 e·Å^–3^

**Table S2.** Selected bond lengths (Å) and angles (deg) for **Pt-Me-impy**.

_____________________________________________________

Pt(1)-C(22) 1.960(5)

Pt(1)-C(19) 2.008(5)

Pt(1)-C(28) 2.034(4)

Pt(1)-N(2) 2.107(4)

C(22)-Pt(1)-C(19) 89.7(2)

C(22)-Pt(1)-C(28) 80.0(2)

C(19)-Pt(1)-C(28) 164.65(19)

C(22)-Pt(1)-N(2) 169.10(16)

C(19)-Pt(1)-N(2) 89.73(18)

C(28)-Pt(1)-N(2) 102.55(17)

C(3)-N(2)-Pt(1) 115.1(3)

C(7)-N(2)-Pt(1) 127.3(3)

C(18)-C(19)-Pt(1) 126.0(4)

C(20)-C(19)-Pt(1) 119.7(4)

C(23)-C(22)-Pt(1) 128.3(4)

C(27)-C(22)-Pt(1) 117.2(4)

N(29)-C(28)-Pt(1) 113.8(3)

N(36)-C(28)-Pt(1) 139.1(4)

C(82)-Pt(61)-C(79) 89.7(2)

C(82)-Pt(61)-C(88) 80.45(19)

C(79)-Pt(61)-C(88) 165.02(19)

C(82)-Pt(61)-N(62) 167.33(17)

C(79)-Pt(61)-N(62) 89.87(18)

C(88)-Pt(61)-N(62) 102.23(16)

C(83)-C(82)-Pt(61) 127.8(4)

C(87)-C(82)-Pt(61) 116.4(3)

N(96)-C(88)-Pt(61) 140.1(3)

N(89)-C(88)-Pt(61) 113.8(3)

_____________________________________________________________

Symmetry transformations used to generate equivalent atoms:

**Table S3. Photophysical and electrochemical properties of BD-02.**

|  | UV–Vis^(a)^  (nm) | λ_RT/77 K_^(b)^  (nm) | FWHM_RT/77 K_^(b)^  (nm) | E_T1_  (eV) | E_g_^(c)^  (eV) | HOMO/LUMO^(d)^  (eV) |
| --- | --- | --- | --- | --- | --- | --- |
| BD-02 | 316;367 | 452 / 445 | 36 / 15 | 2.79 | 2.89 | −5.62/−2.73 |

^(a)^MC solution (1.0 × 10^−5^ M). ^(b)^MC solution (1.0 × 10^−5^ M) at RT and THF solution (1.0 × 10^−5^ M) at 77 K. ^(c)^Optical energy gaps were estimated from the onset wavelengths of the UV–Vis absorption spectra. ^(d)^HOMO levels were determined from the onset of the oxidation curves, while LUMO levels were calculated using the HOMO levels and E_g_.

**Table S4**. The key parameters of **BD-02**, **Pt-impy**, and **Pt-Me-impy** in the excited state.

|  | T_1_ (eV) | <S_0_|H_SOC_|T_1_> (cm^-1^) | ^3^MLCT (%) | *λ* (cm^-1^) | RMSD | *k_r_* (s^-1^) |
| --- | --- | --- | --- | --- | --- | --- |
| BD-02 | 2.669 | 142.24 | 8.76 | 3793.7 | 0.256 | 9.97×10^5^ |
| Pt-impy | 2.561 | 158.68 | 9.42 | 2839.8 | 0.166 | 1.23×10^6^ |
| Pt-Me-impy | 2.615 | 153.74 | 9.24 | 2754.1 | 0.150 | 1.15×10^6^ |

**Table S5**. The electronic configuration and their corresponding contributions for T_1_ state.

|  | Transition | Coefficient | ^3^MLCT (%) |
| --- | --- | --- | --- |
| **BD-02** | HOMO-2 → LUMO | 0.33936 | 1.4810 |
|  | HOMO-1 → LUMO | 0.12533 | 0.2790 |
|  | HOMO →LUMO | 0.55892 | 7.0038 |
| **Pt-impy** | HOMO-2 → LUMO | 0.32507 | 1.6273 |
|  | HOMO-1 → LUMO | 0.12874 | 0.2861 |
|  | HOMO → LUMO | 0.55756 | 7.1687 |
|  | HOMO → LUMO+2 | 0.10416 | 0.3391 |
| **Pt-Me-impy** | HOMO-2 → LUMO | 0.2925 | 1.4048 |
|  | HOMO-1 → LUMO | 0.1926 | 0.6477 |
|  | HOMO → LUMO | 0.55705 | 6.8826 |
|  | HOMO → LUMO+4 | 0.1178 | 0.3078 |

**Table S6. Device performances of D1 for BD-02.**

| Device  (doping concentration) | V_d_^(a)^  (V) | λ  (nm) | | FWHM  (nm) | | CIE | | | | | EQE  (%) | | | |  |
| --- | --- | --- | --- | --- | --- | --- | --- | --- | --- | --- | --- | --- | --- | --- | --- |
|  |  |  |  |  |  | x | | y | | [1,000 cd/m^2^] | | | [max] | |  |
| D1, BD-02  (3 wt%) | 5.0 | | 457 | | 19 | | 0.14 | | 0.10 | | | 23.3 | | 25.8 | |

**Table S7. Device performance of D2 device for BD-02**

| Device  (doping concentration) | V_d_^(a)^  (V) | λ  (nm) | | FWHM  (nm) | | CIE | | | | | EQE  (%) | | | | LT_95_  (h) | |  |
| --- | --- | --- | --- | --- | --- | --- | --- | --- | --- | --- | --- | --- | --- | --- | --- | --- | --- |
|  |  |  |  |  |  | x | | y | | [1,000 cd/m^2^] | | | [max] | |  |  |  |
| BD-02 (8 wt%) | 3.8 | | 461 | | 25 | | 0.14 | | 0.17 | | | 20.1 | | 21.9 | | 88 | |

**8. ^1^H, ^13^C NMR and HRMS**

**Pt-impy**

**
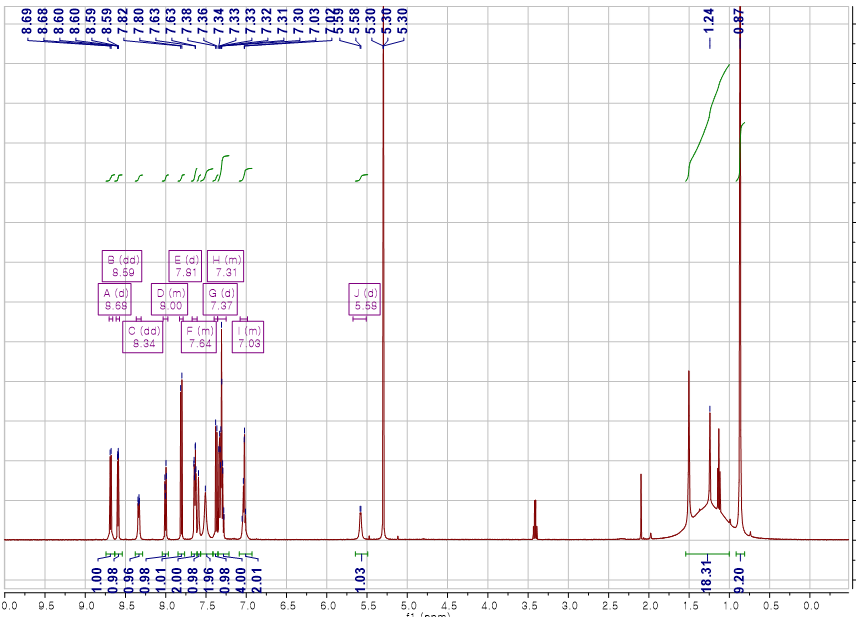
**

^1^H NMR data of **Pt-impy**

**
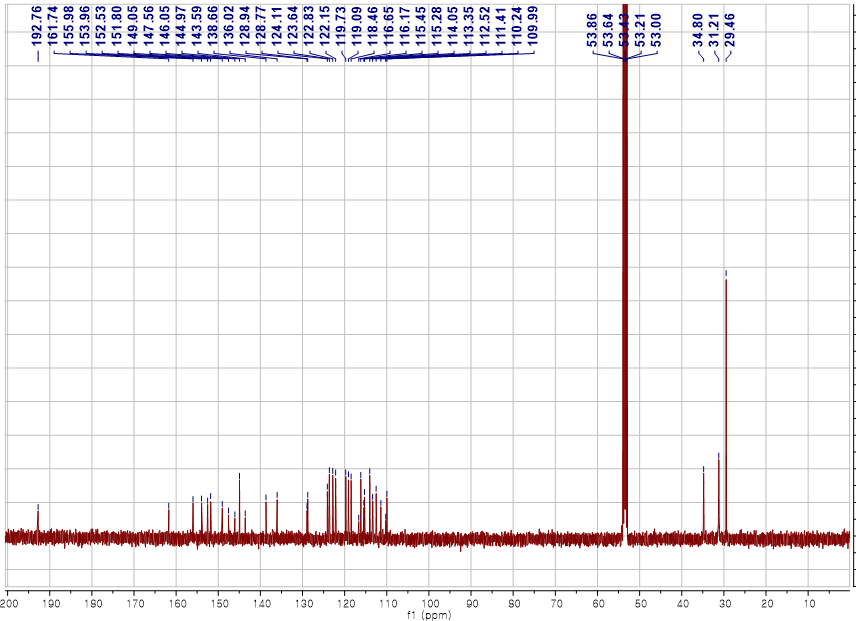
**

^13^C NMR data of **Pt-impy**


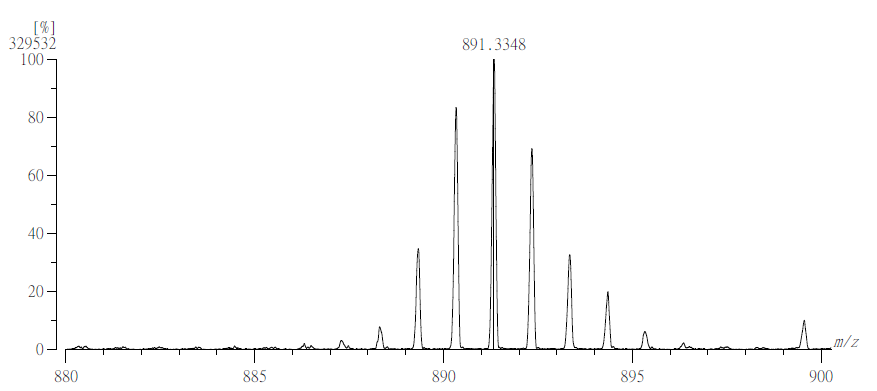


HRMS data of **Pt-impy**

**Pt-Me-impy**

**
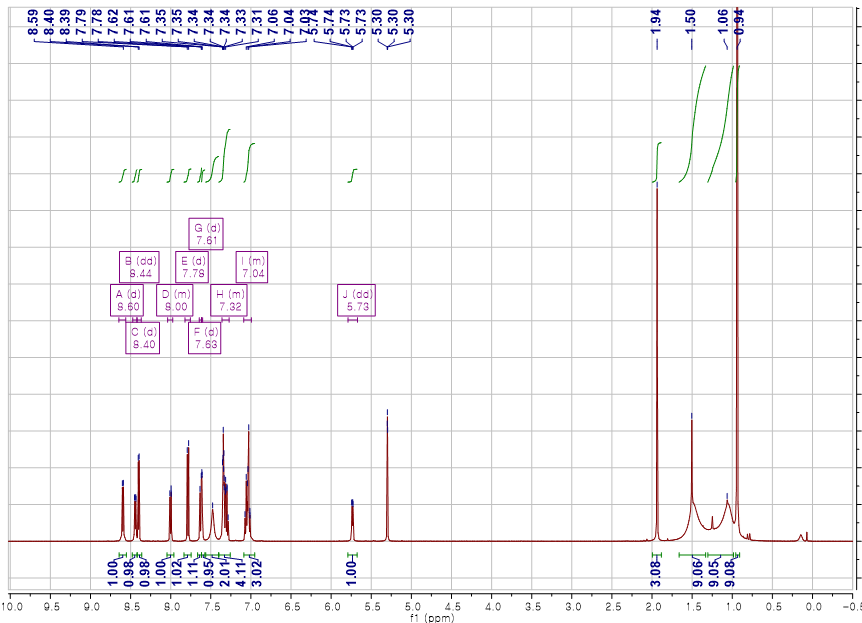
**

^1^H NMR data of **Pt-Me-impy**

**
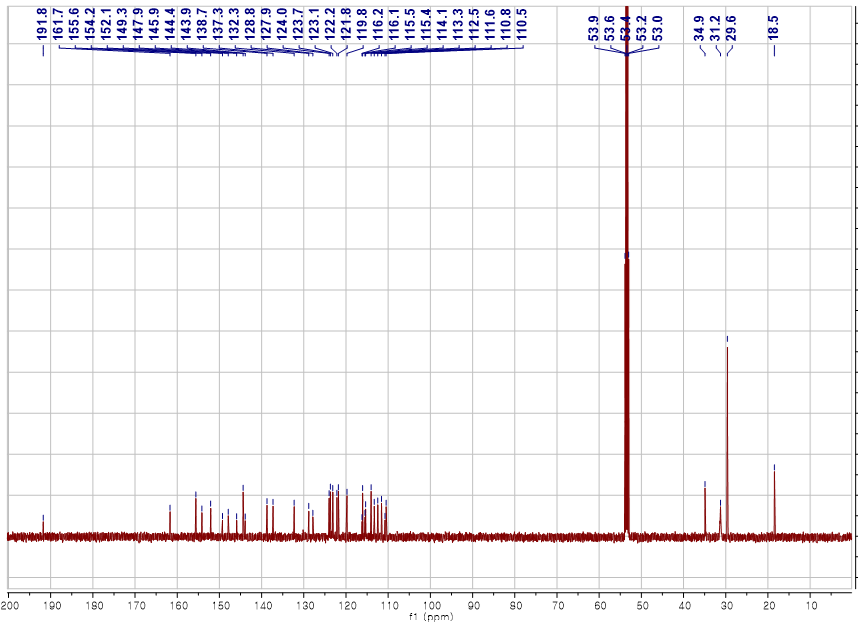
**

^13^C NMR data of **Pt-Me-impy**


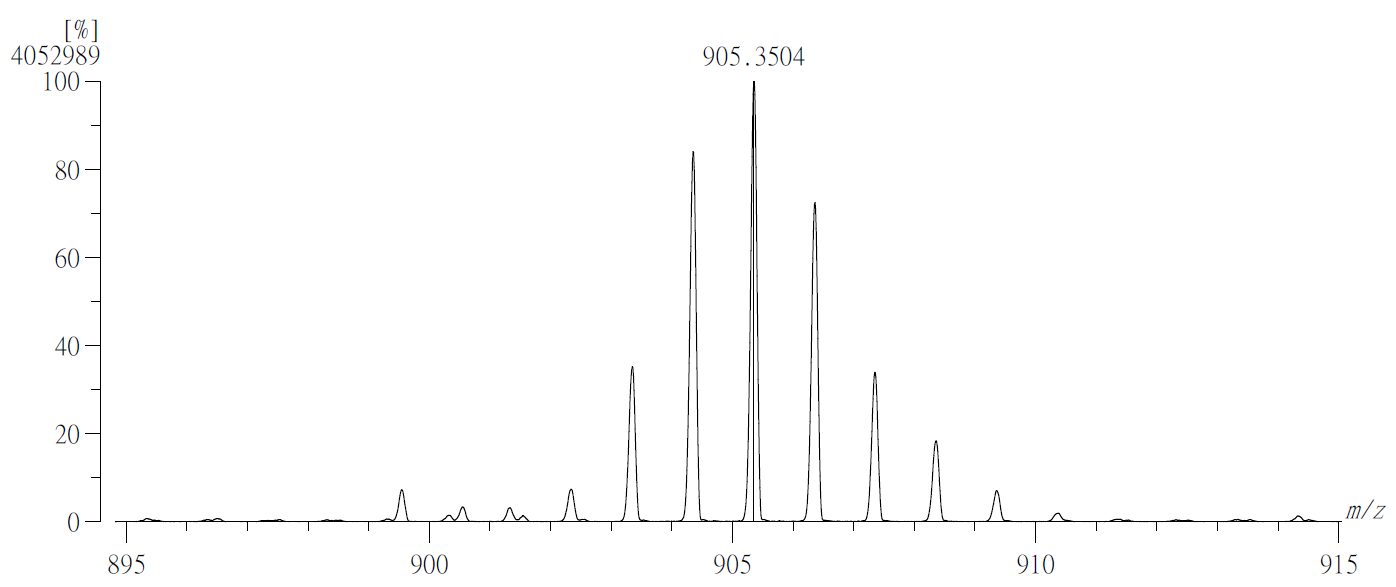


HRMS data of **Pt-Me-impy**

**9. Reference**

[1] M. Frisch, G. Trucks, H. Schlegel, G. Scuseria, M. Robb, J. Cheeseman, G. Scalmani, V. Barone, G. Petersson, H. Nakatsuji, *Gaussian16 (Revision A. 03)* **2016**.

[2] a)A. D. Becke, *J. Chem. Phys.* **1992**, 96, 2155; b)C. Lee, W. Yang, R. G. Parr, *Phys. Rev. B* **1988**, 37, 785; c)S. H. Vosko, L. Wilk, M. Nusair, *Can. J. Phys.* **1980**, 58, 1200; d)P. J. Stephens, F. J. Devlin, C. F. Chabalowski, M. J. Frisch, *J. Phys. Chem.* **1994**, 98, 11623.

[3] G. Li, J. Zheng, X. Fang, K. Xu, Y.-F. Yang, J. Wu, L. Cao, J. Li, Y. She, *Organometallics* **2021**, 40, 472.

[4] J. Sun, H. Ahn, S. Kang, S.-B. Ko, D. Song, H. A. Um, S. Kim, Y. Lee, P. Jeon, S.-H. Hwang, *Nat. Photon.* **2022**, 16, 212.
